# Endometrial Serous Carcinoma: Its Molecular Characteristics and Histology-Specific Treatment Strategies

**DOI:** 10.3390/cancers4030799

**Published:** 2012-08-07

**Authors:** Kentaro Nakayama, Naomi Nakayama, Masako Ishikawa, Kohji Miyazaki

**Affiliations:** 1 Department of Obstetrics and Gynecology, Shimane University School of Medicine, Izumo 6938501, Japan; 2 Department of Biochemistry, Shimane University School of Medicine, Izumo 6938501, Japan

**Keywords:** endometrial serous carcinoma, endometrial carcinoma, Type II endometrial carcinoma, estrogen independent

## Abstract

Endometrial cancer is the fourth most common malignancy in women, with most cases being classified as early stage endometrioid tumors that carry a favorable prognosis. The endometrial serous histological subtype (ESC), however, while only accounting for 10% of all endometrial cancers is responsible for a disproportionate number of deaths. Unlike the estrogen-dependent, well differentiated endometrioid tumors, which are commonly associated with a younger age of onset, ESCs are estrogen-independent and tend to present at an advanced stage and in older women. Treatment for ESC entails aggressive surgery and multimodal adjuvant therapy. In this review, we describe the clinical behavior, molecular aspects, and treatment strategies for ESC.

## 1. Introduction

Endometrial cancer is classified into two subtypes: Type I (endometrioid histology) and Type II (ESC, clear cell histology) [1]. Type I cancers are generally estrogen dependent, low grade, have minimal myometrial invasion and carry a good prognosis. Type II cancers including ESC and clear cell carcinomas, are not associated with increased exposure to estrogen, and carry a poor prognosis. In Japan, the incidence of Type II cancers has increased from 2–3% of all uterine cancers in the early 1990’s to 10% in 2008 [2]. ESC accounts for 3% of all endometrial carcinomas in Japan [2]. In Western countries, although ESC only accounts for 10% of all uterine cancers, it is responsible for 40% of uterine cancer deaths [3]. Historically, endometrial cancer treatment has been dictated by grade and stage; however, it is clear that histology-specific treatment algorithms are also required if the prognosis of ESC is to be improved.

## 2. Clinical Characteristics of Endometrial Serous Carcinoma (ESC)

Endometrial serous adenocarcinoma (ESC) typically arises in postmenopausal women. The tumor bears histological similarity to ovarian serous adenocarcinoma. ESC has a high propensity for early lymphovascular invasion, as well as intraperitoneal and extra-abdominal spread. It commonly presents at an advanced stage. The overall 5-year survival rate is about 30% for all stages and the recurrence rate after surgery is extremely high (50–80%) [4]. Several groups have reported that 50% of ESC show extra-uterine spread at the time of diagnosis [4,5,6,7]. Interestingly, unlike Type I tumors, in which spread to the regional lymph nodes may be predicted by the depth of myometrial invasion and tumor grade, there are no clear predictive factors of extra-uterine disease for ESC. For example, 37–63% of ESC with no myometrial invasion have extrauterine spread [4,6,8], and 38% of ESC confined to an endometrial polyp have extra-uterine spread [8]. ESC sometimes includes endometrial components [9]. Even if in patients with early-stage disease, a trend toward a worse prognosis was found to exist when ESC comprised even 10% of a tumor [9]. Investigation into the treatment of endometrial carcinoma should include and document tumors with any percentage comprised of ESC [9].

## 3. Pathological and Molecular Features of ESC

**Figure 1:**
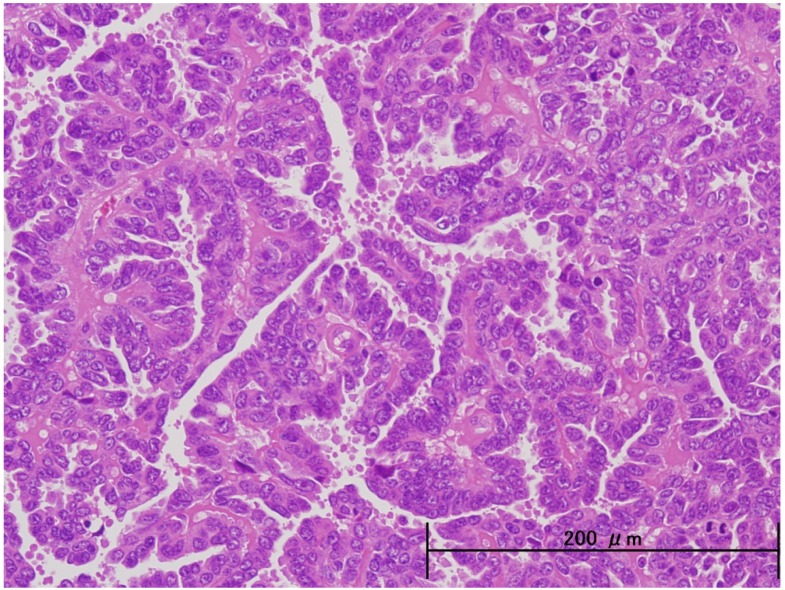
Endometrial serous carcinoma is characterized by high-grade cytological atypia in cells that do not share a common apical border. A papillary architecture is common.

**Figure 2:**
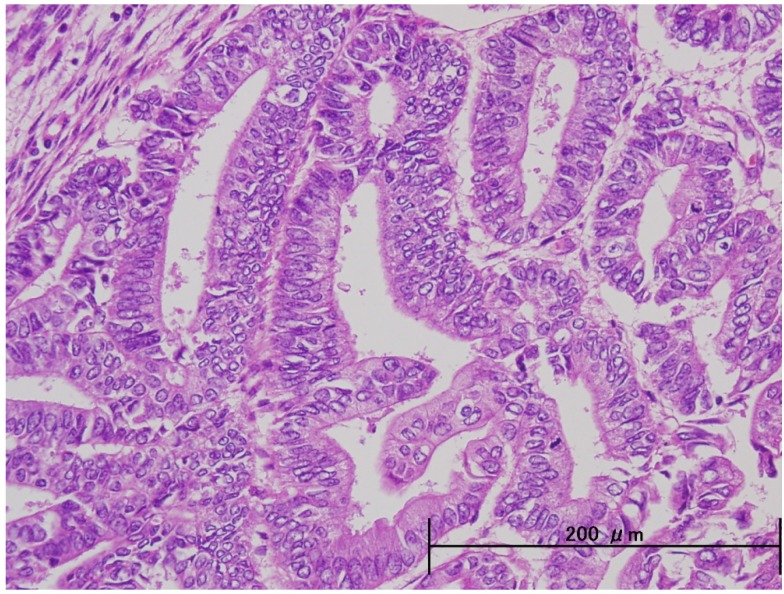
Endometrioid adenocarcinoma, grade 1. The glandular component is a caricature of proliferative phase glands, with stratification and shared luminal border to the neoplastic cells.

Unlike endometrioid adenocarcinomas which arise directly from atypical endometrial hyperplasia, the precursor lesion for ESC, endometrial glandular dysplasia and endometrial intraepithelial carcinoma (EIC), originates in atrophic endometrium [10,11]. ESC closely resembles serous carcinoma of the ovary and fallopian tube because its papillary growth and cellular features are similar (Figure 1). Contrastingly, endmetrioid carcinoma is the most common form of carcinoma of the endometrium, comprising 75% to 80% of the cases. Grade 1 endometrial carcinomas are well differentiated and are generally associated with a good prognosis (Figure 2).

Recently, Zheng *et al.* reported a proposed model of ESC carcinogenesis [11]. According to their model, endometrial serous carcinoma arises predominantly in the resting endometrium, manifesting first as p53 immunoreactive, morphologically normal endometrial cells, evolving to endometrial glandular dysplasia, then to serous endometrial intraepithelial carcinoma (EIC), and finally into fully developed serous carcinoma. Furthermore, they showed that a decreasing percentage of diagnosed lesions express ER, ranging from endometrial glandular dysplasia (EmGD) (70–95%) to serous EIC (<30%) and ESC (<30%) [11]. Loss of ER expression occurred after mutation of *p53* and is associated with HER2/neu overexpression in ESC [11].

ESCs histologically resemble ovarian serous adenocarcinomas. They contain fibrous stroma and show a papillary pattern of proliferation. Tumor cells are pleomorphic, have prominent nuclear atypia, high N/C ratios, distinctive nucleoli, and frequent nuclear fission. Sixty percent of ESCs contain psammoma bodies. Other pathognomonic cellular features include a ballooned or hobnail-like nucleus and stromal hyalinization.

Type I and Type II endometrial carcinomas also differ on a molecular basis. ESCs are not hormone-sensitive and therefore do not express ER or PR [12]. *K-ras*, *PTEN*, *β-catenin*, which are often mutated in Type I endometrioid adenocarcinomas, are rarely altered in ESCs [13]. Instead, mutation of *p53* is often observed [13]. Exomesequencing of ESC identified the frequency of *p53* mutation was 81.6% [14]. Amplification of the HER2/neu gene and overexpression of the HER2/neu protein have been shown in many tumors, including ESC [15,16]. Some studies showed that the overexpression of HER2/neu was frequently associated with the advanced stage and poor prognosis in ESC [15,16,17]. Interestingly, mutation of *PIK3CA* is common in both endometrioid adenocarcinomas and ESCs [18]. Very recently, Kuhn *et al*. reported that amplification of CCNE1 was 30.4% in ESCs [14]. The novel tumor suppressor gene, *ARID1A*, which was recently discovered by an exome sequencing study of ovarian clear cell carcinomas [19,20], is rarely mutated in ESCs. In contrast, *PPP2R1A*, another gene implicated in ovarian clear cell carcinomas, is frequently mutated in ESCs [21,22]. Previously reported clinicopathological and molecular differences between Type I endometrioid adenocarcinomas and Type II ESCs are summarized in Table 1 and Table 2 [23]. Recently, Zhen *et al*. reported that oncofetal protein IMP3 may be a useful diagnostic marker in the assessment of endometrial cancers and their precursor lesions, particularly when the amount available tissue materials is limited and concern of type II cancer arises [24].

**Table 1:** Clinicopathological characteristics of uterine endometrioid and serous carcinomas. *

	Endometrioid	Serous
Precursor lesion	Atypical hyperplasis	Endometrial intraepithelial carcinoma(EIC)
Estrogen	Dependent	Independent
Age	50–60 years	Over 60 years
Menopause	Peri menopause	Post menopausse
Obesity	Positive	Negative
History of Gravidity and Parity	Non	Multi
Grade	Low	High
Myometrial invasion	Slight	Deep
Pattern of recurrence	Local	Distant
Stage of presentation	I (73%)	I (54%)
	II (11%)	II (8%)
	III (13%)	III (22%)
	IV (3%)	IV (16%)
Survival by stage	I (85–90%)	I (50–80%)
	II (70%)	II (50%)
	III (40–50%)	III (20%)
	IV (15–20%)	IV (5–10%)

*: Revised and cited from [22].

**Table 2:** Molecular aspects of uterine endometrioid and serous carcinoma.

	Endometrioid	Serous
Microsatellite instability	30%	0–5%
p53 mutation	10%	90%
K-ras mutation	20–40%	0–5%
ER/PR expression	70–80%	5%
Her-2 amplification/overexpression	15–20%	18–45%
PTEN mutation	40–50%	10%
b-catenin mutation	30%	0–5%
CCNE1 amplification	0–5%	30%
ARID1A mutation	57%	0%
PPP2R1A mutation	5%	41%
PIK3CA mutation	40%	15%

## 4. Therapeutic Strategies for ESC

### 4.1. Surgical Treatment

Historically, the surgical approach for endometrial cancer has not been histology-specific. Deep myometrial invasion is the strongest predictor of extrauterine spread of disease in endometrioid adenocarcinomas and is a factor used to triage patients for lymphadenectomy. Invasion, however, does not predict nodal or intra-abdominal spread in ESC. Therefore, many have advocated extensive staging including pelvic and para-aortic lymphadenectomy and omentectomy even in ESCs with none or minimal myometrial invasion. As in ovarian cancer, optimal cytoreduction has been shown to improve outcomes in stage III and IV ESC [25].

### 4.2. Chemotherapy

The benefit of adjuvant chemotherapy in completely resected stage Ia ESC is controversial; however, the recurrence rate in these patients is 14% without adjuvant treatment [26]. In stage I and II patients with residual disease, the recurrence rate was 43% [27]. Chemotherapy has been shown to have clear benefit in patients with advanced stage disease.

Multiple phase III randomized controlled trials, conducted by the GOG, support the efficacy of systemic chemotherapy in stage III/IV endometrial carcinoma. These regimens are summarized in Table 3. These trials all contained significant numbers of ESC. The response rate to CAP (cyclophosphamide, doxorubicin and cisplatin) ranged from 18–27% [28,29]. The combination of platinum with paclitaxel (T) or docetaxel (D) has been utilized since 2005 in Japan, and has a response rate of about 60% in ESC patients [30,31]. The combination of paclitaxel, doxorubicin and cisplatin (TAP) while effective, was also more toxic than the two-drug regimens [32]. As ESC tends to affect an older population, tolerability is critical. The combination of paclitaxel and carboplatin has recently been shown to be equally efficacious to TAP with minimal toxicity [33].

A randomized phase III study, JGOG2043, is presently underway in Japan. Treatment arms are AP, DP, and TC. The study has reached its quota and is closed to accrual with results pending. Ultimately the results of this study will be used to develop histology-specific treatment protocols.

**Table 3:** Chemotherapy and response rate of serous endometrial cancer.

Regimen	Response rate
AP (Adriamycin + Cisplatin)	42%
AT (Adriamycin + Paclitaxel)	37%
CAP (Cyclophsophamide + Adriamycin + Cisplatin)	18–27%
TAP (Paclitaxel + Adriamycin + Cisplatin)	50%
Paclitaxel + Platinum agent	50–60%

### 4.3. Combination of Chemotherapy and Radiation Therapy

In Europe and the United States, adjuvant pelvic radiotherapy is frequently employed for endometrial cancer patients with lymph node metastasis [4,34]. While adjuvant radiotherapy in early stage (Stages I and II) endometrial cancer has been shown to decrease the rate of pelvic recurrence, a clear survival benefit has not been shown [4,35]. We have evaluated the efficacy of chemotherapy combined with adjuvant radiotherapy [36,37]. Our retrospective study suggested a survival benefit of chemotherapy combined with radiotherapy in patients with stage III/IV disease. As ESCs have a high metastatic potential, it is plausible that chemotherapy combined with radiotherapy may be particularly beneficial in this subtype. However, combination of chemotherapy and radiation therapy, either sequentially or in sandwich fashion for endometrial cancer is still controversial [37].

## 5. Conclusions

ESC is uncommon, but accounts for a disproportionate number of endometrial cancer deaths. Histology-specific treatment algorithms are needed to improve outcomes; however, the development of standardized protocols through randomized controlled trials has been hampered by the rarity of the subtype. Evaluation of the currently available retrospective and prospective data suggests that ESCs benefit from complete cytoreduction followed by adjuvant chemotherapy, regardless of stage. It is hoped that JGOG2043 will address key questions regarding adjuvant chemotherapy. Additional trials are also required to clarify the role of radiotherapy in ESCs.
